# REDUCING HTN AMONG BLACK ELDERS THROUGH AN IMMERSIVE VIRTUAL FOREST

**DOI:** 10.1093/geroni/igad104.2107

**Published:** 2023-12-21

**Authors:** Maureen Mickus

**Affiliations:** Western Michigan University, Kalamazoo, Michigan, United States

## Abstract

Rates of uncontrolled hypertension (HTN) among Black individuals is higher than for any other racial or ethnic group in the U.S. The prevalence of HTN in this minority population, coupled with arterial aging, portend serious risks for cerebrovascular and cardiovascular diseases among Black elders. Past research has indicated that exposure to nature, specifically immersion within a forest setting, can be effective for reducing HTN. But deriving health benefits derived from time in a forest may be difficult for older persons from historically disadvantaged groups. This purpose of this study was to assess whether access to calm and immersive forest images delivered using virtual reality (VR) can modify HTN among community-dwelling Black elders. The study involved a quasi-experimental design and a sample of individuals ages 60-98 recruited from a senior center primarily serving minorities. Each of the 22 participants completed 4 biweekly VR trials using software with a soothing audiovisual simulation of a forest meadow including trees, birds, flowers, and wildlife. Results indicated an average decline of 10 mm HG (SD=7.0) in systolic blood pressure with Hedges g effect sizes ranging from .38 to .96 across sessions. The elders expressed a more relaxed mood and viewed VR as a pleasant distraction overall. Given that none of the participants reported any adverse effects from using this technology, further exploration of forest-based VR as a promising method for decreasing HTN in a high-risk population of older adults is warranted.

